# M. Larrey's Mode of Tapping the Pericardium
*Clinique Chirurgicale, Tome II. p. 303.


**Published:** 1831

**Authors:** 


### M. LARREY'S MODE OF TAPPING THE PERICARDIUM.*

IN hydro-pericarditis, when it is deemed advisable to tap the pericardium, M. Larrey selects the point between the base of the xiphoid cartilage on the left side, and the united extremities of the seventh and eighth ribs on the same side. In this triangular space we may boldly make an incision, extending from the junction of the cartilage of the seventh rib with the sternum, along its inferior border, to the extremity of the cartilage of the eighth, which is closely united to the seventh. In this incision some of the fibres of the rectus and external oblique and cellular tissue are divided, when the knife comes down upon the projecting surface of the pericardium, traversing the triangular space between the two first indigitations of the diaphragm. The point of the knife must then be carefully directed a little upwards and from right to left, in order to open the pericardium without wounding the peritoneum. A small portion of the anterior edge of the diaphragm, at the point of its attachment to the posterior border of the cartilage of the seventh rib, is slightly wounded, but no vessel of any consequence is injured. The heart is less likely to be cut in opening the pericardium in this situation than in any other, because the fluid will naturally collect most at the most dependent part of the bag containing it. M. Larrey, it appears, has not per-

* Clinique Chirurgicale, Tome II. p. 303.

formed the operation, in this situation, on the living body; it is accomplished with great facility on the dead.

---

XLI.

Lithotomy in the Female.

